# Eradication of HIV-1 from the Macrophage Reservoir: An Uncertain Goal?

**DOI:** 10.3390/v7041578

**Published:** 2015-03-31

**Authors:** Wasim Abbas, Muhammad Tariq, Mazhar Iqbal, Amit Kumar, Georges Herbein

**Affiliations:** 1 Department of Biology, SBA School of Science and Engineering, Lahore University of Management Sciences, Lahore 54792, Pakistan; E-Mails: wazim_cemb@hotmail.com (W.A.); m.tariq@lums.edu.pk (M.T.); 2 Laboratory of Drug Discovery and Structural Biology, Health Biotechnology Division, National Institute for Biotechnology and Genetic Engineering (NIBGE), Faisalabad 38000, Pakistan; E-Mail: hamzamgondal@gmail.com; 3 Department of Virology, University of Franche-Comté, CHRU Besançon, UPRES EA4266 Pathogens and Inflammation, SFR FED 4234, 25030 Besançon, France; E-Mail: amit.aiims2005@gmail.com

**Keywords:** HIV-1, cART, latency, reservoirs, macrophage

## Abstract

Human immunodeficiency virus type 1 (HIV-1) establishes latency in resting memory CD4+ T cells and cells of myeloid lineage. In contrast to the T cells, cells of myeloid lineage are resistant to the HIV-1 induced cytopathic effect. Cells of myeloid lineage including macrophages are present in anatomical sanctuaries making them a difficult drug target. In addition, the long life span of macrophages as compared to the CD4+ T cells make them important viral reservoirs in infected individuals especially in the late stage of viral infection where CD4+ T cells are largely depleted. In the past decade, HIV-1 persistence in resting CD4+ T cells has gained considerable attention. It is currently believed that rebound viremia following cessation of combination anti-retroviral therapy (cART) originates from this source. However, the clinical relevance of this reservoir has been questioned. It is suggested that the resting CD4+ T cells are only one source of residual viremia and other viral reservoirs such as tissue macrophages should be seriously considered. In the present review we will discuss how macrophages contribute to the development of long-lived latent reservoirs and how macrophages can be used as a therapeutic target in eradicating latent reservoir.

## 1. Introduction

More than 35 million people have been infected with human immunodeficiency virus type-1 (HIV-1) worldwide [1,2]. With the introduction of combination anti-retroviral therapy (cART) in 1996 HIV-1 infection has become treatable but yet not curable [3,4,5,6,7]. Today, more than 30 different antiretroviral drugs have been approved for HIV treatment [2,8]. These drugs drive the viral load down to undetectable levels. However, the persistence of latent reservoirs of replication-competent non-induced proviruses remains a major obstacle in HIV-1 eradication [3,9,10,11,12,13,14,15,16]. These latent reservoirs are established early during acute viral infection [17,18,19]. Macrophages and latently infected resting CD4+ T cells are reservoirs of HIV-1 [20,21,22,23]. These reservoirs are fully capable of producing infectious viral particles when cART is discontinued [11,15,19,24]. 

Based on the integration status of HIV-1 proviral DNA into the host chromatin, latency has been classified as pre and post integration latency [25,26,27,28]. The role of unintegrated forms of HIV-1 DNA in the formation of viral reservoir is not well established. However, tissue specific cells retain these forms for a longer period of time [29,30]. Post-integration latency occurs when a provirus fails to adequately express its genome and becomes reversibly silenced after integration into the host genome. This latent state is exceptionally stable and mechanisms that maintain HIV-1 latency *in vivo* are not fully understood. Several factors contribute to the silencing of integrated HIV-1 provirus such as the site and orientation of integration into the host genome. These factors include the absence of crucial inducible host factors, the presence of transcriptional repressors, the chromatin structure and epigenetic control of HIV-1 promoter, sequestration of cellular positive transcription factors and the suboptimal concentration of viral transactivators, and inhibition of HIV-1 translation by microRNAs [15,31,32,33,34,35,36]. Most of these mechanisms have been elucidated using transformed cell lines and recently developed primary cell models of HIV-1 latency. However, the relative importance of each mechanism in maintaining viral latency *in vivo* is not fully established.

Reports suggest the HIV-1 infection of circulating monocytes *in vivo.* The infected monocytes can cross the blood-tissue barrier and can differentiate into macrophages [18,26,37,38,39]. Moreover, HIV-1 infected macrophages release several immunoregulatory and inflammatory cytokines including TNF-α, interleukin (IL)-1, and IL-7, which in turn influence viral replication and disease associated with viral infection [40,41]. The successful blockade of HIV-1 replication by cART has shifted the medical research from developing novel antiretroviral drugs towards the eradication of viral reservoirs. A better understanding in the formation of HIV-1 reservoirs will be necessary to uncover the novel targets and methods for purging or eradicating the latent reservoirs. The purpose of this review is to precisely define the viral reservoirs for therapeutic applications.

## 2. HIV-1 Infection of Monocytes/Macrophages

Macrophages play a crucial role in the initial infection, and contribute to HIV-1 pathogenesis throughout the course of viral infection. Since macrophages are an important part of innate immunity and participate indirectly to the adaptive immunity to clear the infection, this makes them a central target of HIV-1 [37,42,43,44,45,46,47,48,49,50]. HIV-1 targets the monocyte/macrophage lineage at varying stages of differentiation [48,49]. For instance data suggests the involvement of a particular monocyte subtype in HIV-1 infection [51]. Phenotypical comparative studies demonstrate that CD14^++^CD16^+^ monocytes are more permissive to productive HIV-1 infection and harbor HIV-1 in infected individuals on cART as compare to the majority of blood monocytes (CD14^++^CD16^−^). In healthy individuals, the CD14^++^CD16^+^ monocytes represent 10% of circulating monocytes [52]. The characteristics have been studied in rhesus macaques. In acute infection, there was an increase in CD14^++^CD16^+^ and CD14^+^CD16^++^ monocytes, while CD14^++^CD16^−^ monocytes decreased two weeks after infection [53]. Similarly, there was increase in CD14^++^CD16^+^ and CD14^+^CD16^++^ monocytes subsets in rhesus macaques with chronic infection and high viral load [53,54]. Moreover, in HIV-1 infected patients, the preferential expansion of CD14^++^CD16^+^ monocyte subset is associated with increased intracellular level of CCL2 [55]. CCL-2 is an important pro-inflammatory chemokine produced during HIV-1 infection and is one of the key factors responsible for the chronic inflammation and tissue damage in HIV-infected patients [56]. For instance, Cinque and colleagues reported a positive correlation between the levels of CCL2 in cerebrospinal fluid of patients with the severity of HIV-1 encephalitis [57]. In another instance, role of CCL-2 has been shown in enhancing the replication of HIV-1 in PBMCs isolated from patients [58]. These monocyte subsets (CD14^++^CD16^+^ and CD14^+^CD16^++^) have been also reported in HCV infection demonstrating that CD16^+^ monocytes may play important role in viral diseases [59,60].

### 2.1. Activation Status of Macrophages and HIV-1 Infection

**Figure 1:**
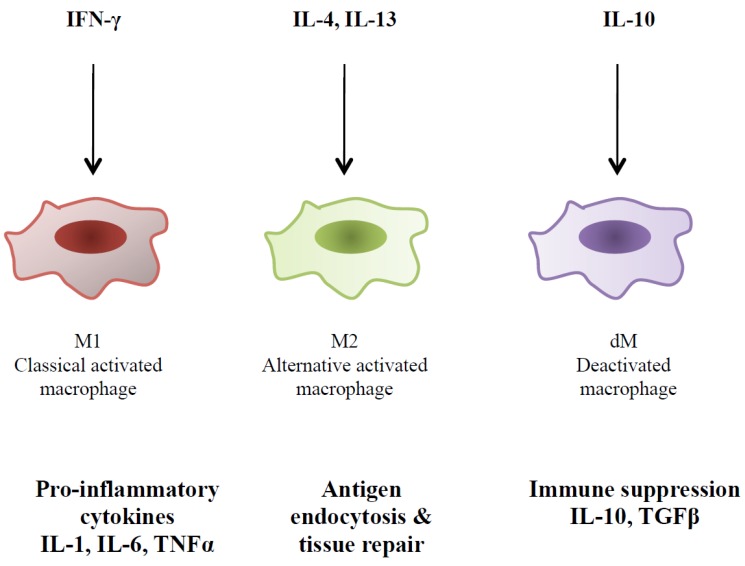
Modulation of macrophage activity by cytokines. Classical activation of macrophages by IFN-γ which display pro-inflammatory characteristics while the alternative activation is mediated by IL-4 and IL-13 and express anti-inflammatory or tissue repairing properties. Macrophages can be deactivated by IL-10.

Monocyte derived macrophages exhibits two distinct types of polarization states depending upon the presence or absence of specific microenvironment stimuli including cytokines. Interestingly, these cytokines also govern HIV-1 pathogenesis. These activation states (classically activated (M1) and alternatively activated macrophages (M2)) play an important role in mediating an effective immune response against infectious agents including HIV-1 [61,62,63,64,65] (Figure 1). The M1 macrophages are activated by a high amount of Th1 cytokines (IFN-γ, IL-2, IL-12), pro-inflammatory cytokines (TNF-α, IL-1β, IL-6, IL-18) and chemokines (CCL3, CCL4, CCL5) that enhance viral replication and block viral entry to prevent superinfection in infected macrophages [64] (Figure 1). M1 macrophages express classical pro-inflammatory cytokines such as TNF-α while M2 macrophages produce anti-inflammatory cytokines such as IL-4, TGF-β and IL-10 by a high amount [62]. During early stages of infection, the M1 macrophages are predominant which cause the tissue injury specifically in lymph nodes that is correlated with T cell apoptosis [66]. However, at later stages of viral infection, there is a shift of M1 to M2 due to the presence of IL-4 and IL-13. The M2 macrophages favor tissue repair and help to clear the opportunistic infections during HIV-1 infection. The progression of HIV-1 infection is accompanied by depletion of CD4+ T cells, resulting in frequent opportunistic infections and the imbalance of Th1 and Th2 responses leads towards the progression of AIDS [64,67]. 

### 2.2. HIV-1 Dynamics in Monocytes/Macrophages: Viral Persistence and Reservoirs

The studies on viral dynamics in monocytes demonstrate that the viral decay in monocytes is slower than that in activated CD4+ T cells. The mean half-life of viral DNA in monocytes/macrophages is longer than that in activated and resting CD4+ T cells suggesting the monocytes/macrophages as an important source of ongoing viral replication in HIV-1-infected patients on cART [68]. Findings suggest that in naïve patients, the activated CD4+ T cells accounts for most of plasma viremia (99%) while the other 1% of the virus may be generated primarily from tissue macrophages [69]. However, in the presence of cART, macrophages are likely the main source of plasma viremia as active viral replication is halted in CD4+ T cells [69,70,71]. Furthermore, it has been reported that circulating monocytes are not a major reservoir of HIV-1 in elite suppressors [72].

### 2.3. Monocytes/Macrophages versus CD4+ T Cells in HIV-1 Infection

Monocyte/macrophages facilitate the transmission and establishment of HIV-1 infection to the CD4+ T cells. Macrophage-tropic HIV-1 variants have been detected during all stages of HIV-1 infection [73]. The chemokine receptor CCR5 is the principal coreceptor for macrophage-tropic HIV-1 on CD4+ T cells and monocytes/macrophages. Several macrophage-tropic variants such as HIV-1_BAL_ (lung macrophages), HIV-1_JR-FL_ (isolated from brain tissue), and HIV-1_Ada_ (from PBMCs) have been isolated from HIV-1 infected patients [74,75,76]. Several studies have demonstrated that monocytes contain HIV-1 variants that are genetically distinct from those observed in CD4+ T cells. Furthermore, the HIV isolates present in monocytes/macrophages are genetically identical or closely associated with viral variants found in the blood of suppressive cART-treated patients for longer periods of time [77,78]. Furthermore, phenotypic studies show that HIV-1 in circulating blood monocytes represents diverse viral phenotypes with multiple coreceptor and cell tropism usage during HIV-1 infection [79,80]. 

It is worth mentioning that opportunistic pathogens such as *Mycobacterium avium* and *Pneumocystis carinii* activate the macrophages and induce HIV production from infected macrophages in lymph nodes [81,82]. These findings suggest that macrophages can be a prominent source of viremia at later stages of HIV when lymphoid tissues are quantitatively and qualitatively impaired and opportunistic pathogens fuel HIV pathogenesis by activating and increasing the viral production from infected macrophages [40,71,81,82]. In addition, T cells also induce HIV-1 replication in myeloid cells. For example, HIV-1 replication in J22-HL-60 (promonocytic cell line) has been reported following direct contact with MOLT-4 T cells, providing the insight into the molecular mechanisms that regulate virus production from monocytes/macrophages which are latently infected with HIV-1 [83]. Moreover, macrophages selectively capture and engulf virally infected CD4+ T cells, a phenomenon that may contribute to the formation or persistence of viral reservoirs [84,85]. 

## 3. Modulation of Macrophage Biology by HIV-1

The life span of macrophages varies greatly and depends upon their tissue location. The tissue macrophages are long lived with a half-life of six weeks to several years. The cells of monocyte-macrophage lineage are highly resistant to viral cytopathic effects and apoptosis, and exhibit longer life spans even when they are exposed to different oxidative stress stimuli [86,87,88]. The macrophages of central nervous system such as microglia and perivascular macrophages produce and release toxins that induce apoptosis of neurons and astrocytes, contributing to the HIV-1-associated dementia [87,88,89,90,91]. 

**Figure 2:**
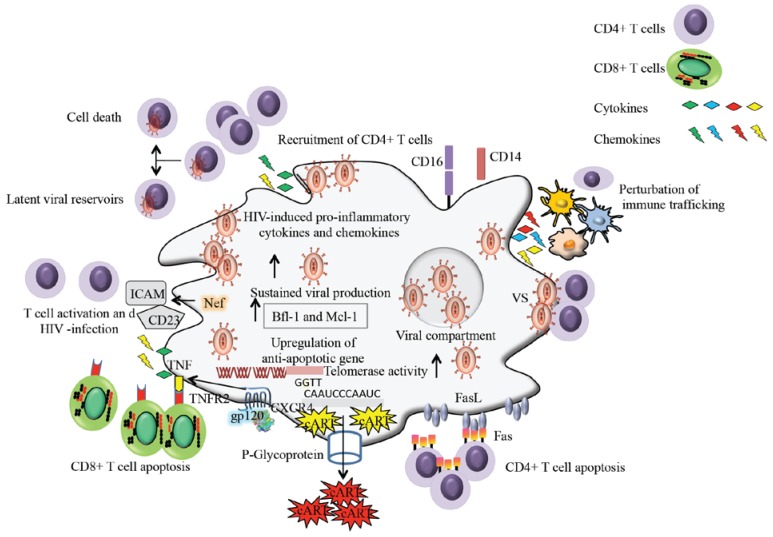
Macrophages fuel HIV-1 pathogenesis. HIV-1 infected macrophages secrete pro-inflammatory cytokines and chemokines that attract T cells in their vicinity, thereby transmitting virus to uninfected CD4+ T cells. Infected CD4+ T cells die soon (due to viral cytopathic effects or antiviral immune response) or return into memory CD4+ T cells as latent viral reservoirs. HIV-1 infected macrophages secrete soluble CD23 and ICAM that results in CD4+ T cell activation favoring the viral infection to CD4+ T cells. Viral gp120 increases the expression of TNF-α and TNFR2 in macrophages and T cells, resulting in CD8+ T cell apoptosis. Bystander CD4+ T cell apoptosis is triggered by FasL ligation to Fas receptor. HIV-1 infection of macrophages enhances its telomerase activity. HIV-1 expands macrophage survival by upregulating antiapoptotic genes. The P-glycoprotein transporter present on macrophages pumps out the antiretroviral drugs and limits the distribution of antiretroviral drugs to macrophages. Furthermore, macrophages spread the virus to CD4+ T cells through virological synapses. HIV-1 infected macrophages store virus into the intracellular cytoplasmic compartments providing the protection against antiviral immune response. HIV-1 infection of macrophages results in the secretion of pro-inflammatory cytokines and chemokines that ultimately accounts for the perturbation of immune trafficking.

It is worth mentioning that HIV-1 infection differentially regulates the telomerase activity in immune cells. Several studies reported that HIV-1 negatively regulates the telomerase activity in CD4+ T cells, CD8+ T cells and Jurkat T cells [92,93]. Furthermore, HIV-1 elite suppressors have longer telomeres and have higher telomerase activity [94]. Interestingly, a study has recently reported that HIV-1 infection of macrophages increases their telomerase activity. The increase in telomerase activity was specific to HIV-1 infection and correlated with p24 antigen production [95,96]. Moreover, increase in telomerase activity by either HIV-1 infection or by overexpression of human telomerase results in higher resistance of macrophages against oxidative stress and DNA damage. Collectively data suggest that HIV-1 infection of macrophages provides better protection against oxidative stress which could be an important viral strategy to make HIV-1-infected macrophages long lived and more resistant viral reservoirs (Figure 2). Furthermore, HIV-1 infection of macrophages favors the expression of macrophage colony stimulating factor (M-CSF) [97]. M-CSF is a prosurvival cytokine that down-regulates TNF-related apoptosis inducing ligand (TRAIL-R1/DR4) and upregulates the anti-apoptotic genes such as Bfl-1 and Mcl-1. Subsequently HIV-1 infected macrophages are resistant to apoptosis induced by TRAIL [97]. 

In addition, HIV-1 infection of macrophages has been shown to modulate apoptosis and promote infection of resting CD4+ T cells. In macrophages, Nef activates a variety of signaling pathways that leads to the infection of bystander CD4+ T cells and hence expands viral reservoirs. Nef-expressing macrophages enhance resting CD4+ T cells infection through multiple cellular and soluble interactions involving macrophages and T cells [40,98]. Nef interacts with apoptosis signal regulating kinase-1 (ASK-1) and inhibits Fas- and TNF receptor-mediated apoptosis in HIV-1-infected CD4+ T cells [40,99]. Reports suggest that the survival of infected CD4+ T cells requires intercellular contacts between macrophages and CD4+ T cells, and expression of Nef [100]. 

On the other hand, HIV-infected macrophages have been shown to induce apoptosis in uninfected CD4+ T and CD8+ T cells. *In vitro* experiments demonstrated that apoptosis inducing ligands expressed by macrophages govern apoptosis of uninfected CD4+ T cells [101,102,103]. The expression of TNF-α and TNFR increases during HIV-1 infection and is associated with the depletion of T cells. Following HIV-1 infection activated macrophages release TNF-α as a soluble factor or expressed as a membrane-bound form that binds to TNFR2. The binding of TNF-α to TNFR2 triggers apoptosis in CD8+ T cells [40,104,105]. In contrast to CD8+ T cells, TNFR2 is not increased on CD4+ T cells, and the apoptosis of CD4+ T cells is mediated through the interaction of Fas and FasL [40,106]. Furthermore, HIV-1 Tat upregulates the production of TRAIL in macrophages and results in the apoptosis of bystander CD4+ T cells [107]. Moreover, the binding of gp120 to CXCR4 upregulates the expression of membrane bound TNF-α and TNFR2 in macrophages and CD8+ T cells respectively (Figure 2). The binding of TNF-α and TNFR2 is associated with decreased intracellular level of Bcl-XL resulting in apoptosis of CD8+ T cells [108]. 

## 4. Macrophages Disseminate HIV-1 to CD4+ T Cells

HIV-1 infected macrophages contribute significantly to the pathogenesis of HIV infection through transmission of virus to CD4+ T cells [42] (Figure 2). It has been reported that HIV-1 infected macrophages fuse and transmit virus to CD4+ T cells through virological synapses [109,110,111,112]. In addition to virological synapses, HIV-1 infected macrophages also secrete viral containing exosomes and microvesicles that facilitate and enhance HIV-1 dissemination to uninfected CD4+ T cells [44]. The production of chemokines by HIV-1-infected monocytes/macrophages favors the recruitment and the activation of a variety of immune cells (Figure 2). *In vitro*, HIV infection of macrophages leads to the production of several chemokines such as CCL-2, CCL-3, CCL-4 and CCL-5 [113,114,115] which in turn favor the recruitment of immune cells including monocytes, macrophages, dendritic cells and T cells. The HIV-1 Nef protein plays a critical role for this function. The adenovirus-mediated expression of Nef in macrophages induces chemokine production that results in chemotaxis and activation of CD4+ T cells for productive HIV-1 infection [116,117,118]. In addition, HIV-1 Nef intersects the macrophage CD40L signaling pathway and promotes the resting CD4+ T cell infection by inducing soluble CD23 and soluble ICAM [119]. 

## 5. Macrophage Infection under cART

The activity of different antiretroviral drugs has been investigated in macrophages chronically infected with HIV-1 [120,121]. Protease inhibitors (PIs) have been shown to be a powerful therapeutic tool to fight HIV infection [122,123]. The combination of PIs along with reverse transcriptase inhibitors has the ability to target the viral replication at early and late stages of HIV infection. The activity of PIs such as saquinavir and ritonavir on HIV-1 infection in monocytes/macrophages was found to be several folds lower than in T cells [120]. Furthermore, the intracellular concentrations of active metabolites of nucleoside analogs were significantly lower (5 to 140 fold) in macrophages than in lymphocytes. The high expression of P-glycoprotein transporter in macrophages has been reported to limit the availability and absorption of these drugs [124,125,126]. This remarkable feature renders the macrophages resistant to certain antiretroviral drugs and ultimately promotes the emergence of viral escape mutants [127,128]. Furthermore, pharmacological inhibition of P-glycoprotein transport enhances absorption and distribution of HIV-1 protease inhibitors to different organs [129,130]. The relatively lower antiviral activity of anti-HIV drugs in macrophages allows continued HIV-1 replication, which may result in the formation of HIV-1 reservoirs and emergence of resistant virus. 

*In situ* hybridization studies on simian immunodeficiency virus HIV type 1 chimera (SHIV) showed that the tissue macrophages in lymph nodes contain high plasma virus in the absence of CD4+ T cells [131]. Quantitative analysis reveals that most of virus producing cells (95%) in these tissues are macrophages and 2% are T lymphocytes. In addition, the administration of potent HIV reverse transcriptase inhibitors blocked the virus production during early infection in T cells but not in macrophages [131]. During macrophage infection, the presence of an individual mutation in HIV integrase is sufficient to produce virus resistant to raltegravir [132]. A recent study by Micci and co-workers demonstrated that the macrophages act as a prominent source of virus in the rhesus macaques that were experimentally depleted of CD4+ T cells followed by SIV infection [133]. Altogether, these different lines of evidence demonstrate that macrophages provide a favorable environment for HIV persistence [133,134]. 

## 6. Cellular Restrictions Factors and HIV Replication in Macrophages 

The importance of macrophages in HIV-1 pathogenesis is further underlined with the discoveries of the presence of anti-HIV-1 cellular restriction factors. Some restriction factors were found to be macrophage-specific and some play role in several cell types. SAMHD1 (sterile alpha motif domain- and HD domain-containing protein 1) is a cellular restriction factor that restricts the replication of HIV-1 and Vpx deficient HIV-2 [135,136]. Noteworthy, SAMHD1 is not specific for macrophages and was initially reported as restriction factor in dendritic cells and apparently also plays a role in CD4+ T cells. SAMHD1 has dNTPase activity that significantly reduces the dNTPs pools, thereby limiting the reverse transcriptase (RT) activity of HIV. VPx protein of HIV-2 has been shown to promote proteasome dependent degradation of SAMHD1 [135]. Despite of absence of Vpx in HIV-1 genome, virus successfully replicates in the macrophages. Recently, Kyei and colleagues reported the direct involvement of cyclin L2 in triggering the proteasomal degradation of SAMHD1 [137]. In addition to SAMHD1, p21 (also called CDKN1A) has been shown to restrict the replication of HIV-1 in MDMs by governing the expression of ribonucleotide reductase subunit R2 [138]. This resulted in the decreased intracellular dNTPs pools limiting the RT activity of HIV-1 [138]. Several other HIV-1 restriction factors have been described including APOBEC3A, APOBEC3G [139,140,141,142,143], tetherin [144,145], TRIM5-alpha [146] and MX2 [147] suggesting the significant importance of macrophages in HIV-1 pathogenesis. 

## 7. Post-Integration Reactivation of HIV from Macrophages 

Post integrated HIV-1 DNA has been well characterized in macrophages at least *in vitro* and to lesser extent *in vivo* [148]. Barr *et al.* sequenced and analyzed 754 unique integration sites in macrophages infected with HIV-1 *in vitro.* They found the preferential integration of HIV-1 in active transcriptional units [149]. HIV-1 was found to be integrated in Toll-like receptor and CAP-binding protein complex interacting homologue genes [150]. The viral replication in monocytes isolated from HIV-1 patients under cART has been reported [151,152]. However, whether HIV-1 was in unintegrated or integrated form was not characterized [152].

**Figure 3:**
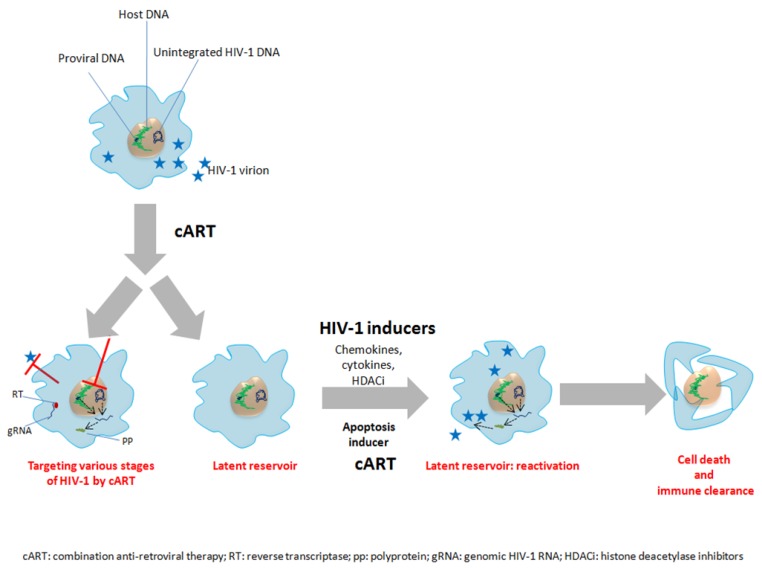
Therapeutic approaches could favor the clearance of HIV-1 from macrophage reservoirs. Macrophages harbor integrated as well as unintegrated proviral DNA. Antiretroviral therapy interferes with several steps of HIV-1 life cycle including entry, reverse transcription, proviral DNA integration, polyprotein processing and release of viral progeny. HIV-1 infection also results in the establishment of latency in less studied reservoirs (macrophages). Macrophages harboring latent HIV-1 [157,158] can be activated by variety of approaches including chemokines, cytokines and HDACi. In addition several apoptotic reagents have been also employed which can specifically induce apoptosis in infected macrophages *in vitro* [44].

Several latently infected cell lines have been routinely used to study the HIV latency, such as U1 cells. Proinflammatory chemokines like TNF alpha and HDAC inhibitors (HDACi) have been found to be effective in reactivating HIV-1 in these model latent cell lines *in vitro* (Figure 3)*.* For instance, HDACi givinostat, belinostat and panobinostat have been shown to decrease the expression of HIV-1 coreceptor CCR5 and to increase viral growth in U1 cells [153]. In another instance the bromodomain inhibitor JQ1 has been shown to reactivate HIV-1 in U1 cells [154]. However, the impact of biological or pharmacological HIV-1 inducers such as HDACi could be difficult to assess in latently infected macrophages. The presence of multidrug pumps in macrophage and inability to reach the tissue specific macrophages in sufficient concentration could contribute to the ineffectiveness of HIV-1 inducers in reactivating HIV-1 in macrophages *in vivo* [155,156]. The study of drugs reactivating HIV from latently infected monocytes/macrophages such as HDACi and bromodomain inhibitors and apoptosis inducing agents [44] need further investigation especially *in vivo* in order to potentially clear HIV-1 from the cellular reservoir in HIV-infected patients (Figure 3). 

## 8. Conclusions

There are several reasons that explain why macrophages play an important role in the pathogenesis of HIV-1. From HIV standpoint, macrophages provide an ideal environment for the formation of viral reservoirs since they live long, are widely distributed throughout the body and are relatively resistant to HIV-induced apoptosis. Moreover, HIV-1 infection enhances the survival of macrophages by upregulating antiapoptotic genes. HIV-1 infection of macrophages activates host transcription factors such as NF-kB and prevents the macrophages from TNF-induced apoptosis. Furthermore, virally infected macrophages secrete CC-chemokines that attract the T lymphocytes in their vicinity leading to their productive viral infection. In addition, activated macrophages could favor the depletion of both uninfected CD4+ T cells and CD8+ T cells leading to immune deficiency. Altogether, macrophages play a critical role in HIV pathogenesis by expanding the viral reservoir that ultimately fuels disease progression. HIV-infected monocytes/macrophages are less sensitive to cART as compared to infected CD4+ T cells. Therefore the development of new therapeutic approaches to clear HIV from monocyte/macrophage reservoirs is under way although total clearance of HIV from macrophage reservoirs is still an uncertain goal that needs to be reached in the future to definitively cure HIV-infected patients. 

## List of Abbreviations

HIV-1: human immunodeficiency virus type-1
TNF-α: tumor necrosis factor alpha
TGF-β: transforming growth factor beta
IL: interleukin
IFN-γ: interferon gamma
cART: combination anti-retroviral therapy
AZT: azidothymidine
TRAIL: TNF-related apoptosis-inducing ligand
ASK-1: apoptosis signal regulating kinase-1
ICAM: intercellular adhesion molecule
SAMHD1: sterile alpha motif domain- and HD domain-containing protein 1
HDAC: histone deacetylase
HDACi: HDAC inhibitor

## References

[1] Maartens G., Celum C., Lewin S.R. (2014). HIV infection: Epidemiology, pathogenesis, treatment, and prevention. Lancet.

[2] Ruelas D.S., Greene W.C. (2013). An integrated overview of HIV-1 latency. Cell.

[3] Anderson J.L., Fromentin R., Corbelli G.M., Østergaard L., Ross A.L. (2015). Progress Towards an HIV Cure: Update from the 2014 International AIDS Society Symposium. AIDS Res. Hum. Retrovirus..

[4] Walensky R.P., Paltiel A.D., Losina E., Mercincavage L.M., Schackman B.R., Sax P.E., Weinstein M.C., Freedberg K.A. (2006). The survival benefits of AIDS treatment in the United States. J. Infect. Dis..

[5] Perelson A.S., Essunger P., Cao Y., Vesanen M., Hurley A., Saksela K., Markowitz M., Ho D.D. (1997). Decay characteristics of HIV-1-infected compartments during combination therapy. Nature.

[6] Murray C.J., Ortblad K.F., Guinovart C., Lim S.S., Wolock T.M., Roberts D.A., Dansereau E.A., Graetz N., Barber R.M., Brown J.C. (2014). Global, regional, and national incidence and mortality for HIV, tuberculosis, and malaria during 1990–2013: A systematic analysis for the Global Burden of Disease Study 2013. Lancet.

[7] Palella F.J., Delaney K.M., Moorman A.C., Loveless M.O., Fuhrer J., Satten G.A., Aschman D.J., Holmberg S.D. (1998). Declining morbidity and mortality among patients with advanced human immunodeficiency virus infection. HIV Outpatient Study Investigators. N. Engl. J. Med..

[8] Passaes C.P., Saez-Cirion A. (2014). HIV cure research: Advances and prospects. Virology.

[9] Ho Y.C., Shan L., Hosmane N.N., Wang J., Laskey S.B., Rosenbloom D.I., Lai J., Blankson J.N., Siliciano J.D., Siliciano R.F. (2013). Replication-competent noninduced proviruses in the latent reservoir increase barrier to HIV-1 cure. Cell.

[10] Margolis D.M. (2014). How might we cure HIV?. Curr. Infect. Dis. Rep..

[11] Deng K., Siliciano R.F. (2014). HIV: Early treatment may not be early enough. Nature.

[12] Katlama C., Deeks S.G., Autran B., Martinez-Picado J., van Lunzen J., Rouzioux C., Miller M., Vella S., Schmitz J.E., Ahlers J. (2013). Barriers to a cure for HIV: New ways to target and eradicate HIV-1 reservoirs. Lancet.

[13] Richman D.D., Margolis D.M., Delaney M., Greene W.C., Hazuda D., Pomerantz R.J. (2009). The challenge of finding a cure for HIV infection. Science.

[14] Archin N.M., Margolis D.M. (2014). Emerging strategies to deplete the HIV reservoir. Curr. Opin. Infect. Dis..

[15] Van Lint C., Bouchat S., Marcello A. (2013). HIV-1 transcription and latency: An update. Retrovirology.

[16] Coiras M., Lopez-Huertas M.R., Perez-Olmeda M., Alcami J. (2009). Understanding HIV-1 latency provides clues for the eradication of long-term reservoirs. Nat. Rev. Microbiol..

[17] Deeks S.G., Lewin S.R., Havlir D.V. (2013). The end of AIDS: HIV infection as a chronic disease. Lancet.

[18] Zaikos T.D., Collins K.L. (2014). Long-lived reservoirs of HIV-1. Trends Microbiol..

[19] Hong F.F., Mellors J.W. (2015). Changes in HIV reservoirs during long-term antiretroviral therapy. Curr. Opin. HIV AIDS.

[20] Svicher V., Ceccherini-Silberstein F., Antinori A., Aquaro S., Perno C.F. (2014). Understanding HIV compartments and reservoirs. Curr. HIV/AIDS Rep..

[21] Alexaki A., Liu Y., Wigdahl B. (2008). Cellular reservoirs of HIV-1 and their role in viral persistence. Curr. HIV Res..

[22] Ananworanich J., Dube K., Chomont N. (2015). How does the timing of antiretroviral therapy initiation in acute infection affect HIV reservoirs?. Curr. Opin. HIV AIDS.

[23] Abbas W., Herbein G. (2012). Molecular understanding of HIV-1 latency. Adv. Virol..

[24] Siliciano R.F. (2014). Opening fronts in HIV vaccine development: Targeting reservoirs to clear and cure. Nat. Med..

[25] Colin L., van Lint C. (2009). Molecular control of HIV-1 postintegration latency: Implications for the development of new therapeutic strategies. Retrovirology.

[26] Kumar A., Abbas W., Herbein G. (2014). HIV-1 latency in monocytes/macrophages. Viruses.

[27] Marcello A. (2006). Latency: The hidden HIV-1 challenge. Retrovirology.

[28] Durand C.M., Blankson J.N., Siliciano R.F. (2012). Developing strategies for HIV-1 eradication. Trends Immunol..

[29] Pang S., Koyanagi Y., Miles S., Wiley C., Vinters H.V., Chen I.S. (1990). High levels of unintegrated HIV-1 DNA in brain tissue of AIDS dementia patients. Nature.

[30] Kelly J., Beddall M.H., Yu D., Iyer S.R., Marsh J.W., Wu Y. (2008). Human macrophages support persistent transcription from unintegrated HIV-1 DNA. Virology.

[31] Margolis D.M. (2010). Mechanisms of HIV latency: An emerging picture of complexity. Curr. HIV/AIDS Rep..

[32] Cherrier T., le Douce V., Eilebrecht S., Riclet R., Marban C., Dequiedt F., Goumon Y., Paillart J.C., Mericskay M., Parlakian A. (2013). CTIP2 is a negative regulator of P-TEFb. Proc. Natl. Acad. Sci. USA.

[33] Eilebrecht S., le Douce V., Riclet R., Targat B., Hallay H., van Driessche B., Schwartz C., Robette G., van Lint C., Rohr O. (2014). HMGA1 recruits CTIP2-repressed P-TEFb to the HIV-1 and cellular target promoters. Nucleic Acids Res..

[34] Eilebrecht S., Schwartz C., Rohr O. (2013). Non-coding RNAs: Novel players in chromatin-regulation during viral latency. Curr. Opin. Virol..

[35] Al-Harthi L., Kashanchi F. (2011). Mechanisms of HIV-1 latency post HAART treatment area. Curr. HIV Res..

[36] Carpio L., Klase Z., Coley W., Guendel I., Choi S., van Duyne R., Narayanan A., Kehn-Hall K., Meijer L., Kashanchi F. (2010). MicroRNA machinery is an integral component of drug-induced transcription inhibition in HIV-1 infection. J. RNAi Gene Silenc..

[37] Le Douce V., Herbein G., Rohr O., Schwartz C. (2010). Molecular mechanisms of HIV-1 persistence in the monocyte-macrophage lineage. Retrovirology.

[38] Smith P.D., Meng G., Salazar-Gonzalez J.F., Shaw G.M. (2003). Macrophage HIV-1 infection and the gastrointestinal tract reservoir. J. Leukoc. Biol..

[39] Veazey R.S., deMaria M., Chalifoux L.V., Shvetz D.E., Pauley D.R., Knight H.L., Rosenzweig M., Johnson R.P., Desrosiers R.C., Lackner A.A. (1998). Gastrointestinal tract as a major site of CD4+ T cell depletion and viral replication in SIV infection. Science.

[40] Herbein G., Gras G., Khan K.A., Abbas W. (2010). Macrophage signaling in HIV-1 infection. Retrovirology.

[41] Kumar A., Abbas W., Herbein G. (2013). TNF and TNF receptor superfamily members in HIV infection: New cellular targets for therapy?. Mediators Inflamm..

[42] Campbell J.H., Hearps A.C., Martin G.E., Williams K.C., Crowe S.M. (2014). The importance of monocytes and macrophages in HIV pathogenesis, treatment, and cure. AIDS.

[43] Watters S.A., Mlcochova P., Gupta R.K. (2013). Macrophages: The neglected barrier to eradication. Curr. Opin. Infect. Dis..

[44] Kumar A., Herbein G. (2014). The macrophage: A therapeutic target in HIV-1 infection. Mol. Cell. Ther..

[45] Zhu T., Muthui D., Holte S., Nickle D., Feng F., Brodie S., Hwangbo Y., Mullins J.I., Corey L. (2002). Evidence for human immunodeficiency virus type 1 replication *in vivo* in CD14+ monocytes and its potential role as a source of virus in patients on highly active antiretroviral therapy. J. Virol..

[46] Lambotte O., Taoufik Y., de Goer M.G., Wallon C., Goujard C., Delfraissy J.F. (2000). Detection of infectious HIV in circulating monocytes from patients on prolonged highly active antiretroviral therapy. J. Acquir. Immune Defic. Syndr..

[47] McElrath M.J., Steinman R.M., Cohn Z.A. (1991). Latent HIV-1 infection in enriched populations of blood monocytes and T cells from seropositive patients. J. Clin. Invest..

[48] Kulkosky J., Bray S. (2006). HAART-persistent HIV-1 latent reservoirs: Their origin, mechanisms of stability and potential strategies for eradication. Curr. HIV Res..

[49] Cribbs S.K., Lennox J., Caliendo A.M., Brown L.A., Guidot D.M. (2015). Healthy HIV-1-infected individuals on highly active antiretroviral therapy harbor HIV-1 in their alveolar macrophages. AIDS Res. Hum. Retrovirus..

[50] Thieblemont N., Weiss L., Sadeghi H.M., Estcourt C., Haeffner-Cavaillon N. (1995). CD14lowCD16high: A cytokine-producing monocyte subset which expands during human immunodeficiency virus infection. Eur. J. Immunol..

[51] Sonza S., Mutimer H.P., Oelrichs R., Jardine D., Harvey K., Dunne A., Purcell D.F., Birch C., Crowe S.M. (2001). Monocytes harbour replication-competent non-latent HIV-1 in patients on highly active antiretroviral therapy. AIDS.

[52] Ellery P.J., Tippett E., Chiu Y.L., Paukovics G., Cameron P.U., Solomon A., Lewin S.R., Gorry P.R., Jaworowski A., Greene W.C. (2007). The CD16+ monocyte subset is more permissive to infection and preferentially harbors HIV-1 *in vivo*. J. Immunol..

[53] Kim W.K., Sun Y., Do H., Autissier P., Halpern E.F., Piatak M., Lifson J.D., Burdo T.H., McGrath M.S., Williams K. (2010). Monocyte heterogeneity underlying phenotypic changes in monocytes according to SIV disease stage. J. Leukoc. Biol..

[54] Crowe S.M., Ziegler-Heitbrock L. (2010). Editorial: Monocyte subpopulations and lentiviral infection. J. Leukoc. Biol..

[55] Ansari A.W., Meyer-Olson D., Schmidt R.E. (2013). Selective expansion of pro-inflammatory chemokine CCL2-loaded CD14+CD16+ monocytes subset in HIV-infected therapy naive individuals. J. Clin. Immunol..

[56] Ansari A.W., Heiken H., Meyer-Olson D., Schmidt R.E. (2011). CCL2: A potential prognostic marker and target of anti-inflammatory strategy in HIV/AIDS pathogenesis. Eur. J. Immunol..

[57] Cinque P., Vago L., Mengozzi M., Torri V., Ceresa D., Vicenzi E., Transidico P., Vagani A., Sozzani S., Mantovani A. (1998). Elevated cerebrospinal fluid levels of monocyte chemotactic protein-1 correlate with HIV-1 encephalitis and local viral replication. AIDS.

[58] Vicenzi E., Alfano M., Ghezzi S., Gatti A., Veglia F., Lazzarin A., Sozzani S., Mantovani A., Poli G. (2000). Divergent regulation of HIV-1 replication in PBMC of infected individuals by CC chemokines: Suppression by RANTES, MIP-1alpha, and MCP-3, and enhancement by MCP-1. J. Leukoc. Biol..

[59] Coquillard G., Patterson B.K. (2009). Determination of hepatitis C virus-infected monocyte lineage reservoirs in individuals with or without HIV coinfection. J. Infect. Dis..

[60] Dichamp I., Abbas W., Kumar A., di Martino V., Herbein G. (2014). Cellular activation and intracellular HCV load in peripheral blood monocytes isolated from HCV monoinfected and HIV-HCV coinfected patients. PLOS ONE.

[61] Mills C.D., Ley K. (2014). M1 and M2 macrophages: The chicken and the egg of immunity. J. Innate Immun..

[62] Xuan W., Qu Q., Zheng B., Xiong S., Fan G.H. (2015). The chemotaxis of M1 and M2 macrophages is regulated by different chemokines. J. Leukoc. Biol..

[63] Cassol E., Cassetta L., Alfano M., Poli G. (2010). Macrophage polarization and HIV-1 infection. J. Leukoc. Biol..

[64] Herbein G., Varin A. (2010). The macrophage in HIV-1 infection: From activation to deactivation?. Retrovirology.

[65] Italiani P., Boraschi D. (2014). From monocytes to M1/M2 macrophages: Phenotypical *versus* functional differentiation. Front. Immunol..

[66] Herbein G., Khan K.A. (2008). Is HIV infection a TNF receptor signalling-driven disease?. Trends Immunol..

[67] Clerici M., Shearer G.M. (1993). A TH1-->TH2 switch is a critical step in the etiology of HIV infection. Immunol. Today.

[68] Crowe S., Zhu T., Muller W.A. (2003). The contribution of monocyte infection and trafficking to viral persistence, and maintenance of the viral reservoir in HIV infection. J. Leukoc. Biol..

[69] Zhu T. (2002). HIV-1 in peripheral blood monocytes: An underrated viral source. J. Antimicrob. Chemother..

[70] Kedzierska K., Crowe S.M. (2002). The role of monocytes and macrophages in the pathogenesis of HIV-1 infection. Curr. Med. Chem..

[71] Stevenson M. (2014). Role of myeloid cells in HIV-1-host interplay. J. Neurovirol..

[72] Spivak A.M., Salgado M., Rabi S.A., O’Connell K.A., Blankson J.N. (2011). Circulating monocytes are not a major reservoir of HIV-1 in elite suppressors. J. Virol..

[73] Schuitemaker H., Kootstra N.A., de Goede R.E., de Wolf F., Miedema F., Tersmette M. (1991). Monocytotropic human immunodeficiency virus type 1 (HIV-1) variants detectable in all stages of HIV-1 infection lack T-cell line tropism and syncytium-inducing ability in primary T-cell culture. J. Virol..

[74] Gendelman H.E., Orenstein J.M., Martin M.A., Ferrua C., Mitra R., Phipps T., Wahl LA., Lane H.C., Fauci A.S., Burke D.S. (1988). Efficient isolation and propagation of human immunodeficiency virus on recombinant colony-stimulating factor 1-treated monocytes. J. Exp. Med..

[75] Gartner S., Markovits P., Markovitz D.M., Betts R.F., Popovic M. (1986). Virus isolation from and identification of HTLV-III/LAV-producing cells in brain tissue from a patient with AIDS. JAMA.

[76] Koyanagi Y., Miles S., Mitsuyasu R.T., Merrill J.E., Vinters H.V., Chen I.S. (1987). Dual infection of the central nervous system by AIDS viruses with distinct cellular tropisms. Science.

[77] Llewellyn N., Zioni R., Zhu H., Andrus T., Xu Y., Corey L., Zhu T. (2006). Continued evolution of HIV-1 circulating in blood monocytes with antiretroviral therapy: Genetic analysis of HIV-1 in monocytes and CD4+ T cells of patients with discontinued therapy. J. Leukoc. Biol..

[78] Fulcher J.A., Hwangbo Y., Zioni R., Nickle D., Lin X., Heath L., Mullins J.I., Corey L., Zhu T. (2004). Compartmentalization of human immunodeficiency virus type 1 between blood monocytes and CD4+ T cells during infection. J. Virol..

[79] Xu Y., Zhu H., Wilcox C.K., van’t Wout A., Andrus T., Llewellyn N., Stamatatos L., Mullins J.I., Corey L., Zhu T. (2008). Blood monocytes harbor HIV type 1 strains with diversified phenotypes including macrophage-specific CCR5 virus. J. Infect. Dis..

[80] Valcour V.G., Shiramizu B.T., Shikuma C.M. (2010). HIV DNA in circulating monocytes as a mechanism to dementia and other HIV complications. J. Leukoc. Biol..

[81] Caselli E., Galvan M., Cassai E., Caruso A., Sighinolfi L., di Luca D. (2005). Human herpesvirus 8 enhances human immunodeficiency virus replication in acutely infected cells and induces reactivation in latently infected cells. Blood.

[82] Orenstein J.M., Fox C., Wahl S.M. (1997). Macrophages as a source of HIV during opportunistic infections. Science.

[83] Qi X., Koya Y., Saitoh T., Saitoh Y., Shimizu S., Ohba K., Yamamoto N., Yamaoka S., Yamamoto N. (2007). Efficient induction of HIV-1 replication in latently infected cells through contact with CD4+ T cells: Involvement of NF-kappaB activation. Virology.

[84] Baxter A.E., Russell R.A., Duncan C.J., Moore M.D., Willberg C.B., Pablos J.L., Finzi A., Kaufmann D.E., Ochsenbauer C., Kappes J.C. (2014). Macrophage infection via selective capture of HIV-1-infected CD4+ T cells. Cell Host Microbe.

[85] Kugelberg E. (2015). Macrophages: Capturing HIV-infected T cells. Nat. Rev. Immunol..

[86] Carter C.A., Ehrlich L.S. (2008). Cell biology of HIV-1 infection of macrophages. Annu. Rev. Microbiol..

[87] Jones G., Power C. (2006). Regulation of neural cell survival by HIV-1 infection. Neurobiol. Dis..

[88] McNelis J.C., Olefsky J.M. (2014). Macrophages, immunity, and metabolic disease. Immunity.

[89] Coleman C.M., Wu L. (2009). HIV interactions with monocytes and dendritic cells: Viral latency and reservoirs. Retrovirology.

[90] Fischer T., Wyatt C.M., D’Agati V.D., Croul S., McCourt L., Morgello S., Rappaport J. (2014). Mononuclear phagocyte accumulation in visceral tissue in HIV encephalitis: Evidence for increased monocyte/macrophage trafficking and altered differentiation. Curr. HIV Res..

[91] Tavazzi E., Morrison D., Sullivan P., Morgello S., Fischer T. (2014). Brain inflammation is a common feature of HIV-infected patients without HIV encephalitis or productive brain infection. Curr. HIV Res..

[92] Reynoso R., Minces L., Salomon H., Quarleri J. (2006). HIV-1 infection downregulates nuclear telomerase activity on lymphoblastoic cells without affecting the enzymatic components at the transcriptional level. AIDS Res. Hum. Retrovirus..

[93] Franzese O., Adamo R., Pollicita M., Comandini A., Laudisi A., Perno C.F., Aquaro S., Bonmassar E. (2007). Telomerase activity, hTERT expression, and phosphorylation are downregulated in CD4+ T lymphocytes infected with human immunodeficiency virus type 1 (HIV-1). J. Med. Virol..

[94] Ballon G., Ometto L., Righetti E., Cattelan A.M., Masiero S., Zanchetta M., Chieco-Bianchi L., de Ross I.A. (2001). Human immunodeficiency virus type 1 modulates telomerase activity in peripheral blood lymphocytes. J. Infect. Dis..

[95] Lichterfeld M., Mou D., Cung T.D., Williams KL., Waring M.T., Huang J., Pereyra F., Trocha A., Freeman G.J., Rosenberg E.S. (2008). Telomerase activity of HIV-1-specific CD8+ T cells: Constitutive up-regulation in controllers and selective increase by blockade of PD ligand 1 in progressors. Blood.

[96] Ojeda D., Lopez-Costa J.J., Sede M., López E.M., Berria M.I., Quarleri J. (2014). Increased *in vitro* glial fibrillary acidic protein expression, telomerase activity, and telomere length after productive human immunodeficiency virus-1 infection in murine astrocytes. J. Neurosci. Res..

[97] Osman A., Bhuyan F., Hashimoto M., Nasser H., Maekawa T., Suzu S. (2014). M-CSF inhibits anti-HIV-1 activity of IL-32, but they enhance M2-like phenotypes of macrophages. J. Immunol..

[98] Swingler S., Brichacek B., Jacque J.M., Ulich C., Zhou J., Stevenson M. (2003). HIV-1 Nef intersects the macrophage CD40L signalling pathway to promote resting-cell infection. Nature.

[99] Geleziunas R., Xu W., Takeda K., Ichijo H., Greene W.C. (2001). HIV-1 Nef inhibits ASK1-dependent death signalling providing a potential mechanism for protecting the infected host cell. Nature.

[100] Mahlknecht U., Deng C., Lu M.C., Greenough T.C., Sullivan J.L., O’Brien W.A., Herbein G. (2000). Resistance to apoptosis in HIV-infected CD4+ T lymphocytes is mediated by macrophages: Role for Nef and immune activation in viral persistence. J. Immunol..

[101] Oyaizu N., McCloskey T.W., Coronesi M., Chirmule N., Kalyanaraman V.S., Pahwa S. (1993). Accelerated apoptosis in peripheral blood mononuclear cells (PBMCs) from human immunodeficiency virus type-1 infected patients and in CD4 cross-linked PBMCs from normal individuals. Blood.

[102] Badley A.D., McElhinny J.A., Leibson P.J., Lynch D.H., Alderson M.R., Paya C.V. (1996). Upregulation of Fas ligand expression by human immunodeficiency virus in human macrophages mediates apoptosis of uninfected T lymphocytes. J. Virol..

[103] Badley A.D., Dockrell D., Simpson M., Schut R., Lynch D.H., Leibson P., Paya C.V. (1997). Macrophage-dependent apoptosis of CD4+ T lymphocytes from HIV-infected individuals is mediated by FasL and tumor necrosis factor. J. Exp. Med..

[104] Grell M., Douni E., Wajant H., Löhden M., Clauss M., Maxeiner B., Georgopoulos S., Lesslauer W., Kollias G., Pfizenmaier K. (1995). The transmembrane form of tumor necrosis factor is the prime activating ligand of the 80 kDa tumor necrosis factor receptor. Cell.

[105] Zheng L., Fisher G., Miller R.E., Peschon J., Lynch D.H., Lenardo M.J. (1995). Induction of apoptosis in mature T cells by tumour necrosis factor. Nature.

[106] Lahdevirta J., Maury C.P., Teppo A.M., Repo H. (1988). Elevated levels of circulating cachectin/tumor necrosis factor in patients with acquired immunodeficiency syndrome. Am. J. Med..

[107] Yang Y., Tikhonov I., Ruckwardt T.J., Djavani M., Zapata J.C., Pauza C.D., Salvato M.S. (2003). Monocytes treated with human immunodeficiency virus Tat kill uninfected CD4+ cells by a tumor necrosis factor-related apoptosis-induced ligand-mediated mechanism. J. Virol..

[108] Herbein G., Mahlknecht U., Batliwalla F., Gregersen P., Pappas T., Butler J., O’Brien W.A., Verdin E. (1998). Apoptosis of CD8+ T cells is mediated by macrophages through interaction of HIV gp120 with chemokine receptor CXCR4. Nature.

[109] Crowe S.M., Mills J., Elbeik T., Lifson J.D., Kosek J., Marshall J.A., Engleman E.G., McGrath M.S. (1992). Human immunodeficiency virus-infected monocyte-derived macrophages express surface gp120 and fuse with CD4 lymphoid cells *in vitro*: A possible mechanism of T lymphocyte depletion *in vivo*. Clin. Immunol. Immunopathol..

[110] Crowe S.M., Mills J., Kirihara J., Boothman J., Marshall J.A., McGrath M.S. (1990). Full-length recombinant CD4 and recombinant gp120 inhibit fusion between HIV infected macrophages and uninfected CD4-expressing T-lymphoblastoid cells. AIDS Res. Hum. Retrovirus..

[111] Peressin M., Proust A., Schmidt S., Su B., Lambotin M., Biedma M.E., Laumond G., Decoville T., Holl V., Moog C. (2014). Efficient transfer of HIV-1 in *trans* and in *cis* from Langerhans dendritic cells and macrophages to autologous T lymphocytes. AIDS.

[112] Duncan C.J., Williams J.P., Schiffner T., Gärtner K., Ochsenbauer C., Kappes J., Russell R.A., Frater J., Sattentau Q.J. (2014). High-multiplicity HIV-1 infection and neutralizing antibody evasion mediated by the macrophage-T cell virological synapse. J. Virol..

[113] Fantuzzi L., Belardelli F., Gessani S. (2003). Monocyte/macrophage-derived CC chemokines and their modulation by HIV-1 and cytokines: A complex network of interactions influencing viral replication and AIDS pathogenesis. J. Leukoc. Biol..

[114] Mengozzi M., de Filippi C., Transidico P., Biswas P., Cota M., Ghezzi S., Vicenzi E., Mantovani A., Sozzani S., Poli G. (1999). Human immunodeficiency virus replication induces monocyte chemotactic protein-1 in human macrophages and U937 promonocytic cells. Blood.

[115] Schmidtmayerova H., Nottet H.S., Nuovo G., Raabe T., Flanagan C.R., Dubrovsky L., Gendelman H.E., Cerami A., Bukrinsky M., Sherry B. (1996). Human immunodeficiency virus type 1 infection alters chemokine beta peptide expression in human monocytes: Implications for recruitment of leukocytes into brain and lymph nodes. Proc. Natl. Acad. Sci. USA.

[116] Swingler S., Mann A., Jacque J., Brichacek B., Sasseville V.G., Williams K., Lackner A.A., Janoff E.N., Wang R., Fisher D. (1999). HIV-1 Nef mediates lymphocyte chemotaxis and activation by infected macrophages. Nat. Med..

[117] Liu X., Shah A., Gangwani M.R., Silverstein P.S., Fu M., Kumar A. (2014). HIV-1 Nef induces CCL5 production in astrocytes through p38-MAPK and PI3K/Akt pathway and utilizes NF-kB, CEBP and AP-1 transcription factors. Sci. Rep..

[118] Verollet C., Souriant S., Bonnaud E., Jolicoeur P., Raynaud-Messina B., Kinnaer C., Fourquaux I., Imle A., Benichou S., Fackler O.T. (2015). HIV-1 reprograms the migration of macrophages. Blood.

[119] Mangino G., Percario Z.A., Fiorucci G., Vaccari G., Acconcia F., Chiarabelli C., Leone S., Noto A., Horenkamp F.A., Manrique S. (2011). HIV-1 Nef induces proinflammatory state in macrophages through its acidic cluster domain: Involvement of TNF alpha receptor associated factor 2. PLOS ONE.

[120] Perno C.F., Newcomb F.M., Davis D.A., Aquaro S., Humphrey R.W., Caliò R., Yarchoan R. (1998). Relative potency of protease inhibitors in monocytes/macrophages acutely and chronically infected with human immunodeficiency virus. J. Infect. Dis..

[121] Gavegnano C., Detorio M.A., Bassit L., Hurwitz S.J., North T.W., Schinazi R.F. (2013). Cellular pharmacology and potency of HIV-1 nucleoside analogs in primary human macrophages. Antimicrob. Agents Chemother..

[122] McQuade T.J., Tomasselli A.G., Liu L., Karacostas V., Moss B., Sawyer T.K., Heinrikson R.L., Tarpley W.G. (1990). A synthetic HIV-1 protease inhibitor with antiviral activity arrests HIV-like particle maturation. Science.

[123] Adachi M., Ohhara T., Kurihara K., Tamada T., Honjo E., Okazaki N., Arai S., Shoyama Y., Kimura K., Matsumura H. (2009). Structure of HIV-1 protease in complex with potent inhibitor KNI-272 determined by high-resolution X-ray and neutron crystallography. Proc. Natl. Acad. Sci. USA.

[124] Kim R.B., Fromm M.F., Wandel C., Leake B., Wood A.J., Roden D.M., Wilkinson G.R. (1998). The drug transporter P-glycoprotein limits oral absorption and brain entry of HIV-1 protease inhibitors. J. Clin. Invest..

[125] Zastre J.A., Chan G.N., Ronaldson P.T., Ramaswamy M., Couraud P.O., Romero I.A., Weksler B., Bendayan M., Bendayan R. (2009). Up-regulation of P-glycoprotein by HIV protease inhibitors in a human brain microvessel endothelial cell line. J. Neurosci. Res..

[126] Robillard K.R., Chan G.N., Zhang G., la Porte C., Cameron W., Bendayan R. (2014). Role of P-glycoprotein in the distribution of the HIV protease inhibitor atazanavir in the brain and male genital tract. Antimicrob. Agents Chemother..

[127] Srinivas R.V., Middlemas D., Flynn P., Fridland A. (1998). Human immunodeficiency virus protease inhibitors serve as substrates for multidrug transporter proteins MDR1 and MRP1 but retain antiviral efficacy in cell lines expressing these transporters. Antimicrob. Agents Chemother..

[128] Jorajuria S., Dereuddre-Bosquet N., Becher F., Martin S., Porcheray F., Garrigues A., Mabondzo A., Benech H., Grassi J., Orlowski S. (2004). ATP binding cassette multidrug transporters limit the anti-HIV activity of zidovudine and indinavir in infected human macrophages. Antivir. Ther..

[129] Choo E.F., Leake B., Wandel C., Imamura H., Wood A.J., Wilkinson G.R., Kim R.B. (2000). Pharmacological inhibition of P-glycoprotein transport enhances the distribution of HIV-1 protease inhibitors into brain and testes. Drug Metab. Dispos..

[130] Zha W., Wang G., Xu W., Liu X., Wang Y., Zha B.S., Shi J., Zhao Q., Gerk P.M., Studer E. (2013). Inhibition of P-glycoprotein by HIV protease inhibitors increases intracellular accumulation of berberine in murine and human macrophages. PLOS ONE.

[131] Igarashi T., Brown C.R., Endo Y., Buckler-White A., Plishka R., Bischofberger N., Hirsch V., Martin M.A. (2001). Macrophage are the principal reservoir and sustain high virus loads in rhesus macaques after the depletion of CD4+ T cells by a highly pathogenic simian immunodeficiency virus/HIV type 1 chimera (SHIV): Implications for HIV-1 infections of humans. Proc. Natl. Acad. Sci. USA.

[132] Marsden M.D., Avancena P., Kitchen C.M., Hubbard T., Zack J.A. (2011). Single mutations in HIV integrase confer high-level resistance to raltegravir in primary human macrophages. Antimicrob. Agents Chemother..

[133] Micci L., Alvarez X., Iriele R.I., Ortiz A.M., Ryan E.S., McGary C.S., Deleage C., McAtee B.B., He T., Apetrei C. (2014). CD4 depletion in SIV-infected macaques results in macrophage and microglia infection with rapid turnover of infected cells. PLOS Pathog..

[134] Adamson C.S., Freed E.O. (2010). Novel approaches to inhibiting HIV-1 replication. Antiviral Res..

[135] Hrecka K., Hao C., Gierszewska M., Swanson S.K., Kesik-Brodacka M., Srivastava S., Srivastava L., Washburn M.P., Washburn J. (2011). Vpx relieves inhibition of HIV-1 infection of macrophages mediated by the SAMHD1 protein. Nature.

[136] Lahouassa H., Daddacha W., Hofmann H., Ayinde D., Logue E.C., Dragin L., Bloch N., Maudet C., Bertrand M., Gramberg T. (2012). SAMHD1 restricts the replication of human immunodeficiency virus type 1 by depleting the intracellular pool of deoxynucleoside triphosphates. Nat. Immunol..

[137] Kyei G.B., Cheng X., Ramani R., Ratner L. (2015). Cyclin L2 is a critical HIV dependency factor in macrophages that controls SAMHD1 abundance. Cell Host Microbe.

[138] Allouch A., David A., Amie S.M., Lahouassa H., Chartier L., Margottin-Goguet F., Barré-Sinoussi F., Kim B., Sáez-Cirión A., Pancino G. (2013). p21-mediated RNR2 repression restricts HIV-1 replication in macrophages by inhibiting dNTP biosynthesis pathway. Proc. Natl. Acad. Sci. USA.

[139] Sheehy A.M., Gaddis N.C., Choi J.D., Malim M.H. (2002). Isolation of a human gene that inhibits HIV-1 infection and is suppressed by the viral Vif protein. Nature.

[140] Peng G., Lei K.J., Jin W., Greenwell-Wild T., Wahl S.M. (2006). Induction of APOBEC3 family proteins, a defensive maneuver underlying interferon-induced anti-HIV-1 activity. J. Exp. Med..

[141] Berger G., Durand S., Fargier G., Nguyen X.N., Cordeil S., Bouaziz S., Muriaux D., Darlix J.L., Cimarelli A. (2011). APOBEC3A is a specific inhibitor of the early phases of HIV-1 infection in myeloid cells. PLOS Pathog..

[142] Sabbatucci M., Covino D.A., Purificato C., Mallano A., Federico M., Lu J., Rinaldi A.O., Pellegrini M., Bona R., Michelini Z. (2015). Endogenous CCL2 neutralization restricts HIV-1 replication in primary human macrophages by inhibiting viral DNA accumulation. Retrovirology.

[143] Vicenzi E., Poli G. (2013). Novel factors interfering with human immunodeficiency virus-type 1 replication *in vivo* and *in vitro*. Tissue Antigens.

[144] Neil S.J., Zang T., Bieniasz P.D. (2008). Tetherin inhibits retrovirus release and isantagonized by HIV-1 Vpu. Nature.

[145] Van Damme N., Goff D., Katsura C., Jorgenson R.L., Mitchell R., Johnson M.C., Stephens E.B., Guatelli J. (2008). The interferon-induced protein BST-2 restricts HIV-1 releaseand is downregulated from the cell surface by the viral Vpu protein. Cell Host Microbe.

[146] Stremlau M., Owens C.M., Perron M.J., Kiessling M., Autissier P., Sodroski J. (2004). The cytoplasmic body component TRIM5a restricts HIV-1infection in old world monkeys. Nature.

[147] Goujon C., Moncorgé O., Bauby H., Doyle T., Ward C.C., Schaller T., Hué S., Barclay W.S., Schulz R., Malim M.H. (2013). Human MX2 is an interferon-induced post-entry inhibitor of HIV-1 infection. Nature.

[148] Siliciano R.F., Greene W.C. (2011). HIV latency. Cold Spring Harb. Perspect. Med..

[149] Barr S.D., Ciuffi A., Leipzig J., Shinn P., Ecker J.R., Bushman F.D. (2006). HIV integration site selection: Targeting in macrophages and the effects of different routes of viral entry. Mol. Ther..

[150] Killebrew D.A., Troelstrup D., Shiramizu B. (2004). Preferential HIV-1 integration sites in macrophages and HIV-associated malignancies. Cell. Mol. Biol..

[151] Arfi V., Riviere L., Jarrosson-Wuilleme L., Goujon C., Rigal D., Darlix J.L., Cimarelli A. (2008). Characterization of the early steps of infection of primary blood monocytes by human immunodeficiency virus type 1. J. Virol..

[152] Harrold S.M., Wang G., McMahon D.K., Riddler S.A., Mellors J.W., Becker J.T., Caldararo R., Reinhart T.A., Achim C.L., Wiley C.A. (2002). Recovery of replication-competent HIV type 1-infected circulating monocytes from individuals receiving antiretroviral therapy. AIDS Res. Hum. Retrovirus..

[153] Rasmussen T.A., Schmeltz Søgaard O., Brinkmann C., Wightman F., Lewin S.R., Melchjorsen J., Dinarello C., Østergaard L., Tolstrup M. (2013). Comparison of HDAC inhibitors in clinical development: Effect on HIV production in latently infected cells and T-cell activation. Hum. Vaccin. Immunother..

[154] Banerjee C., Archin N., Michaels D., Belkina A.C., Denis G.V., Bradner J., Sebastiani P., Margolis D.M., Montano M. (2012). BET bromodomain inhibition as a novel strategy for reactivation of HIV-1. J. Leukoc. Biol..

[155] Kraft-Terry S.D., Stothert A.R., Buch S., Gendelman H.E. (2010). HIV-1 neuroimmunity in the era of antiretroviral therapy. Neurobiol. Dis..

[156] Koppensteiner H., Brack-Werner R., Schindler M. (2012). Macrophages and their relevance in human immunodeficiency virus type 1 infection. Retrovirology.

[157] Montaner L.J., Griffin P., Gordon S. (1994). Interleukin 10 inhibits initial reverse transcription of human immunodeficiency virus type 1 and mediates a virostatic latent state in primary blood-derived human macrophages *in vitro*. J. Gen. Virol..

[158] Brown A., Zhang H., Lopez P., Pardo C.A., Gartner S. (2006). *In vitro* modeling of the HIV-macrophage reservoir. J. Leukoc. Biol..

